# A Sensor Fusion Approach to Observe Quadrotor Velocity

**DOI:** 10.3390/s24113605

**Published:** 2024-06-03

**Authors:** José Ramón Meza-Ibarra, Joaquín Martínez-Ulloa, Luis Alfonso Moreno-Pacheco, Hugo Rodríguez-Cortés

**Affiliations:** 1 Sección de Estudios de Posgrado, Escuela Superior de Ingeniería Mecánica y Eléctrica, Instituto Politécnico Nacional, Av. Instituto Politécnico Nacional, S/N, Col. Lindavista, Ciudad de México 07738, Mexico; jmezai1600@alumno.ipn.mx (J.R.M.-I.); jmartinezu1901@alumno.ipn.mx (J.M.-U.); 2 Departamento de Ingeniería Eléctrica y Electrónica, Instituto Tecnológico Autónomo de México, Río Hondo 1, Tizapán, Mexico City 01080, Mexico; hugo.rodriguez@itam.mx

**Keywords:** visual odometry, optical flow, sensor fusion, observer design, velocity observation

## Abstract

The growing use of Unmanned Aerial Vehicles (UAVs) raises the need to improve their autonomous navigation capabilities. Visual odometry allows for dispensing positioning systems, such as GPS, especially on indoor flights. This paper reports an effort toward UAV autonomous navigation by proposing a translational velocity observer based on inertial and visual measurements for a quadrotor. The proposed observer complementarily fuses available measurements from different domains and is synthesized following the Immersion and Invariance observer design technique. A formal Lyapunov-based observer error convergence to zero is provided. The proposed observer algorithm is evaluated using numerical simulations in the Parrot Mambo Minidrone App from Simulink-Matlab.

## 1. Introduction

Thanks to the advancement of unmanned aerial vehicles, these have gained ground in various applications, including law enforcement, precision agriculture, and architectural/industrial inspection. Additionally, researchers have chosen to use this aircraft type, which offers excellent academic advantages, to test new nonlinear control theory methods. Crewless aircraft have made a quantum leap, and the nonlinear control theory has evolved. The basis of the classical nonlinear control theory, built by, for instance, [1,2], has been adapted rapidly into this century’s new technologies, as reported in [3,4]. Hence, quadrotor aerial vehicles have become a worldwide standard platform for robotics research. In particular, the Parrot Mambo Minidrone App in Simulink-Matlab offers excellent functionality for testing new control and state observation algorithms. It provides semi-realistic inertial and visual sensors, making real-time implementation straightforward. In reference to the first developments of sensor fusion for unmanned aerial vehicles, we can find that in [5], the measurements from an Inertial Navigation System (INS) and GPS sensors are fused by using a Kalman filter. The information for the simulation experiment was used to examine the data of both sensors; tool implementations of the filter were considered. The research article [6] establishes a basic requirement for an autonomous robot merging the data of different sensors; odometric and sonar sensors are fused together by means of an Extended Kalman Filter (EKF). The adaptive algorithm performs a precise localization of the vehicle and can be established in a wide range of experimental situations. The researchers in [7] describe a simple yet powerful statistical technique for fusing information from different sensors that aims to obtain the spectral error densities of the navigation gravity model, completing a robust aerial model that absorbs vertical disturbances. The information is computed in the time-dependent frequency.

In [8], the authors present a real-time implementation of position controllers based on the Adaptive–Proportional–Integral Derivative (APID) method for the Parrot Mambo Minidrone. An adaptive mechanism based on a second-order sliding mode control is also included to modify the conventional parameters of the altitude controller. Simulations and experimental flights validate the success of this approach. The research reported in [9] synthesizes Proportional–Integral–Derivative (PID), Linear Quadratic Regulator (LQR), and Model Predictive Control (MPC) algorithms for a Parrot Minidrone, and the control algorithms’ strengths and weaknesses are analyzed. These control techniques were chosen considering that the MPC design for Parrot Minidrones is unavailable in the existing literature. The automatic code generation capabilities offered by the Simulink coder facilitated the experimental implementation of the proposed control algorithms. The use of a quadrotor linear model evidently causes a relatively large mismatch between simulations and experimental results. The work in [10] presented the real-time implementation of a novel Cartesian translational robust control strategy for the Parrot Mambo Minidrone. Multiple data were collected from actual flight experiments involving a set of four Parrot Mambo Minidrones. A first-order dynamic with time delay was identified as the mathematical model for the Cartesian translational dynamic. The control strategy was designed based on a parameter variant discrete-time linear system. Reference [11] reports the design and implementation of a cascade attitude controller for the Parrot Mambo Minidrone. The controller was designed using the triple-step and nonlinear integral sliding mode control NISMC method considering the three-DOF nonlinear model. In [12], a hand gesture-based drone controller for the Parrot Mambo Minidrone is developed; the controller can perform take-off, hovering, and landing operations. The delay between executing each command is only three seconds, and the system accuracy obtained for hand gesture detection is remarkably good. The system can be further extended by modifying the controller so that the UAV can move and perform multiple commands using hand gestures.

Current directions aim to develop nonlinear observer strategies for autonomous navigation using only onboard sensors. The work in [13] proposed an embedded fast Nonlinear Predictive Model (NMPC) algorithm. This controller ensures a stable and safe flight of micro aerial robots relying solely on onboard sensors to localize themselves. The implemented controller can drive the micro aerial vehicle to track a path and outstand external disturbances. The design of a computationally efficient optical flow algorithm for tiny multirotor aerial vehicles, also known as pocket drones, is the focus of the work reported [14]. The Edge-Flow algorithm uses a compressed representation of an image frame to match it with the one in the previous time step; the adaptive time horizon also enables it to detect sub-pixel flow, from which velocities can be observed. Reference [15] presents a fusion filter design using the Kalman Filter (KF) complemented with the No Motion No Integration Filter (NMNI) to reduce noise influence to observe the tilt angle using only one accelerometer and one gyroscope. This approach relieves the burden of multiple sensors for attitude tracking, reducing battery energy consumption and helping the drone obtain the precise angle under rotor vibrations.

Translational velocity observation for quadrotors is an appealing problem from practical and theoretical perspectives. In [16], a globally exponential convergent observer based on nonlinear adaptive techniques is proposed. The observer uses measurements from an Attitude and Heading Reference System (AHRS). Numerical simulations evaluate the observer algorithm that shows good performance in the presence of noisy acceleration measurements. Reference [17] reports the design of a deterministic quadrotor translational velocity observer employed to reconstruct the scale factor of the position determined by a Simultaneous Localization And Mapping (SLAM) algorithm. The translational velocity observer uses the translational acceleration and the non-scaled translational position; its performance is evaluated through numerical simulations. Translational velocity observation is also an essential issue for fixed-wing aircrafts. Reference [18] presents an exponentially stable nonlinear wind velocity observer for fixed-wing unmanned aerial vehicles. The proposed observer employs a GNSS-aided Inertial Navigation System (INS), an attitude observer, and a pitot-static probe measuring dynamic pressure and airspeed in the longitudinal direction. The proposed observer estimates wind velocity and, as by-products, the angles of attack, sideslip, and scaling factor of the pitot-static probe measurement without requiring UAV maneuvers with the Persistence of Excitation (PE). The algorithm is well-suited for embedded systems and was tested through simulations. Finally, in [19], a uniform, semi-global, exponentially stable nonlinear observer for attitude, gyro bias, position, velocity, and specific force estimation for a fixed-wing UAV is presented. The nonlinear observer uses inertial and visual measurements without any assumptions about the flight altitude or the structure of the terrain being recorded. Experimental data from a UAV test flight and simulated data show that the nonlinear observer performs robustly.

Examining the existing literature reveals a significant gap in quadrotor translational velocity observations. No deterministic observer complements inertial and visual measurements with a formal proof of convergence of the observation error and a semi-realistic numerical simulation. By incorporating novel methodologies such as the Immersion and Invariance method, this research fills these gaps and presents a technique to complementarily fuse both measurements, accompanied by a formal proof of the convergence to zero of the observation error. This research was driven by an aim to enhance quadrotor capabilities to fly autonomously using only onboard sensors. In relation to the autonomous performance of aerial vehicles, we can find in [20], mounted-based stations, which are expected to become an integral component of future intelligent transportation systems. The aerial vehicle, integrated with the ground vehicular network, is used to bridge coverage gaps, offer broader communication services, and improve the network connection stability. Its self understanding is the main task, which is performed by the autonomous unmanned vehicle. As mentioned, a fundamental goal of the autonomous UAV is the development of navigation systems that help to realize any kind of aerial operations; inside [21], a nano-UAV learns to detect and fly through programmed trajectories with an autonomous navigation system based on neural networks. This research article is extremely pretentious because, besides the autonomous requirements, it is needed to establish appropriate sensor fusion, as well as the overall perspective of the whole aerial environment. Key insights from this research include the complementary nature of the inertial and visual sensors. Moreover, the findings reported in this article complement the work presented in [17]. The nonlinear translational velocity observer of [17] degrades when the quadrotor remains in hover; this problem is overcome by adding the optical flow measurement. Adding the optical measurement also gives the proposed observer an advantage over the observer reported in [16]. Finally, it is important to observe that the nonlinear observers reported in [18,19] use different sensors.

This paper introduces a novel sensor fusion strategy for observing the quadrotor translational velocity. The proposed observer algorithm uniquely fuses inertial and visual measurements. This fusion strategy is synthesized following the Immersion and Invariance approach method introduced in [3]. The observer error convergence to zero is guaranteed using Lyapunov theory arguments, and its performance is evaluated through numerical simulations in the semi-realistic Parrot Minidrone app from Matlab-Simulink. It is demonstrated that the proposed observer operates effectively under noisy measurements and considering different quadrotor motions.

This work has the following structure: Section 2 presents the quadrotor model and describes the available measurements. Section 3 reports the sensor fusion algorithm and formally states the nonlinear observer design. Section 4 is devoted to the numerical simulation study, the observation error, and the stability properties of the designed observer; finally, Section 5 presents the conclusions of this work.

## 2. Quadrotor Mathematical Model and Available Measurements

### 2.1. Quadrotor Dynamics

**Figure 1:**
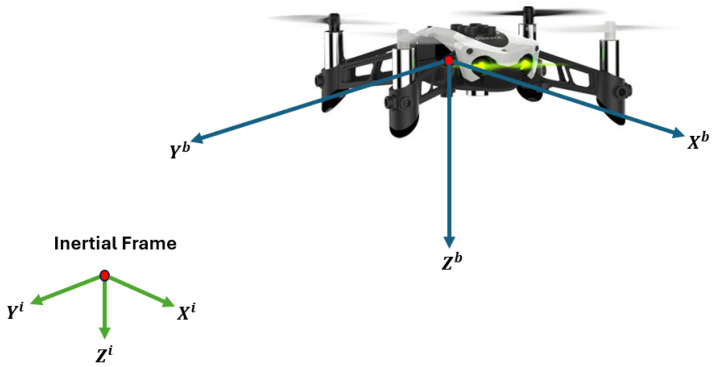
Mambo Parrot’s coordinate frames.

The basic structure of the dynamic quadrotor model has been reported in various research papers and books. This work considers the dynamic quadrotor model reported in [17,22]. The model contains states expressed in two frames of reference, the inertial and the fixed body, and is described by the following set of differential equations. See Figure 1.

(1) $$\begin{array}{cc} \dot{X} & =\;RV^{b}\\ m{\dot{V}}^{b} & =\;mgR^{⊤}e_{3}-T_{T}e_{3}-\mu HV^{b}-mS\left(\Omega \right)V^{b}\\ \dot{R} & =\;RS(\Omega )\\ J\dot{\Omega } & =\;-S\left(\Omega \right)J\Omega +M^{b} \end{array}$$

where $X={\left[\begin{array}{ccc} x & y & z \end{array}\right]}^{⊤}$ is the quarotor position in the inertial reference frame. R∈SO(3) is the rotation matrix from the fixed body to inertial coordinates with

$$SO\left(3\right)=\left\{R\;\in \;R^{3\times 3}\;|\;R^{⊤}R=I,\;\det\left(R\right)=1\right\}$$

where $I\in R^{3\times 3}$ is the identity matrix, and $V^{b}={\left[\begin{array}{ccc} u & v & w \end{array}\right]}^{⊤}$ denotes the translational velocity expressed in the fixed body frame. Moreover, *m* represents the vehicle’s mass, *g* is the gravitational force constant, $e_{3}={\left[0\;0\;1\right]}^{⊤}$, $T_{T}$ is the total thrust force produced by the four rotors,

$$H=\left[\begin{array}{ccc} 1 & 0 & 0\\ 0 & 1 & 0\\ 0 & 0 & 0 \end{array}\right],$$

and S(·) is a skew symmetric matrix such that

$$a\times b=S\left(a\right)b,\;\forall \;a,b\in R^{3},$$

$J=\mathrm{diag}\{J_{xx},J_{yy},J_{zz}\}$ is the inertia matrix, $\Omega ={\left[\begin{array}{ccc} p & q & r \end{array}\right]}^{⊤}$ is the quadrotor rotational velocity expressed in the fixed-body frame coordinates, and $M^{b}$ is the vector of moments generated by the differential rotor’s thrust and rotor’s reaction moment.

### 2.2. Available Measurements

This work considers that the quadrotor has an Inertial Measurement Unit (IMU), a monocular camera pointing downwards, and a height sensor. Thus, the following signals are available.

#### 2.2.1. Quadrotor’s Specific Translational Acceleration

As the leading electronic sensor, multi-rotors carry an IMU, which provides information on the fixed body coordinates. An IMU delivers the specific translational acceleration $a^{b}$, the angular velocity Ω, and the intensity of the Earth’s magnetic field. According to the work reported in [23], the specific translational acceleration measured by the IMU’s accelerometer mounted on board an aerial vehicle is given by

(2) $$a^{b}=\frac{1}{m}F_{T}^{b}-gR^{⊤}e_{3}$$

where $F_{T}^{b}$ models the total external forces acting on the vehicle where the IMU is mounted. From the second equation in (1), it follows that

(3) $$F_{T}^{b}=mgR^{T}e_{3}-T_{T}e_{3}-\mu HV^{b}$$

Notice that the above equation does not include the term $-mS\left(\Omega \right)V^{b}$ because the Coriolis acceleration is an inertial force. Substituting (3) into (2), the specific acceleration force measured by an accelerometer onboard a quadrotor is

(4) $$a^{b}=-\frac{T_{T}}{m}e_{3}-\frac{\mu }{m}HV^{b}$$

The first available measurement is the specific translational acceleration, which element-to-element reads in the following way,

(5) $$y_{1}=a^{b}=\left[\begin{array}{c} a_{x}^{b}\\ a_{y}^{b}\\ a_{z}^{b} \end{array}\right]=-\frac{1}{m}\left[\begin{array}{c} \mu u\\ \mu v\\ T_{T} \end{array}\right]$$

#### 2.2.2. Quadrotor’s Attitude and Angular Velocity

The IMU data processed through an estimation algorithm constitutes an Attitude and Heading Reference System (AHRS) that provides the quadrotor attitude *R* and angular velocity Ω. The AHRS can deliver the attitude using a parameterization such as Euler angles or quaternions or directly delivering the rotation matrix *R*. Thus, one has

(6) $$\begin{array}{ccc} y_{2} & = & R\\ y_{3} & = & \Omega \end{array}$$

#### 2.2.3. Optical Flow

Nowadays, the second crucial sensor for autonomous navigation is a monocular camera. The pixel velocity can be measured and related to the quadrotor velocity by processing the camera image.

The cornerstone to interpreting an image inside a computer is brightness. The camera sensors integrate the irradiance from the scene so that $I(x_{p},y_{p})$ defines the brightness of the pixel located at $\left(x_{p},y_{p}\right)$; thus, the *k* image captured at time *t* can be characterized as [24],

$$I_{k}\left(x_{p},y_{p},t\right):R\subset R^{2}\times R\to R_{+}$$

Computer vision deals with extracting meaningful information from images; this is extracting meaningful information from brightness. A much more straightforward problem is computing image motion information from brightness. Consider two images of the same pixel location, $I_{1}\left(x_{p},y_{p},t_{1}\right)$ and $I_{2}\left(x_{p},y_{p},t_{2}\right)$, taken from infinitesimally close vantage points and at infinitesimally consecutive time instants $t_{2}\approx t_{1}$; thus,

$$\begin{array}{ccc} I_{1}\left(x_{p}\left(t_{1}\right),y_{p}\left(t_{1}\right),t_{1}\right) & = & I_{1}\left(x_{p}\left(t_{1}\right),y_{p}\left(t_{1}\right),t_{1}\right)\\ I_{2}\left(x_{p}\left(t_{2}\right),y_{p}\left(t_{2}\right),t_{2}\right) & = & I_{2}\left(x_{p}\left(t_{1}+\Delta t\right),y_{p}\left(t_{1}+\Delta t\right),t_{1}+\Delta t\right) \end{array}$$

where $t_{2}=t_{1}+\Delta t$ with Δt an infinitesimal time increment. Assume that only pixels belonging to flat and parallel image plane portions and moving parallel to the image plane are considered. Then,

$$\begin{array}{ccc} x_{p}\left(t_{1}+\Delta t\right) & = & x_{p}\left(t_{1}\right)+u_{p}\Delta t\\ y_{p}\left(t_{1}+\Delta t\right) & = & y_{p}\left(t_{1}\right)+v_{p}\Delta t \end{array}$$

with $u_{p}$ and $v_{p}$ the pixel velocity. As a result,

(7) $$\begin{array}{ccc} I_{1}\left(x_{p}\left(t_{1}\right),y_{p}\left(t_{1}\right),t_{1}\right) & = & I_{1}\left(x_{p}\left(t_{1}\right)+u_{p}\Delta t,y_{p}\left(t_{1}\right)+v_{p}\Delta t,t_{1}+\Delta t\right) \end{array}$$

Expanding the right-hand-side of Equation (7) by the Taylor series, one has

(8) $$\begin{array}{ccc} I_{1}\left(x_{p}\left(t_{1}\right),y_{p}\left(t_{1}\right),t_{1}\right) & = & I_{1}\left(x_{p}\left(t_{1}\right),y_{p}\left(t_{1}\right),t_{1}\right)+\frac{\partial I_{1}}{\partial x_{p}}u_{p}\Delta t+\frac{\partial I_{1}}{\partial y_{p}}v_{p}\Delta t+\frac{\partial I_{1}}{\partial t}\Delta t+O\left(\Delta t^{2}\right) \end{array}$$

Neglecting the high-order terms, it follows that

(9) $$\begin{array}{ccc} \frac{\partial I_{1}}{\partial x_{p}}u_{p}+\frac{\partial I_{1}}{\partial y_{p}}v_{p}+\frac{\partial I_{1}}{\partial t} & = & 0 \end{array}$$

this equation is known as the brightness constancy constraint [24] or the optical flow constraint equation [25,26]. Note that it is impossible to compute the speeds $u_{p}$ and $v_{p}$ perpendicular to the image gradient using the brightness constancy constraint; this drawback is known as the aperture problem [27]. It is necessary to evaluate the brightness constancy constraint in each pixel location belonging to a region where $u_{p}$ and $v_{p}$ can be assumed constant, for example, the window

$$\begin{array}{ccc} W_{i}\left(x_{p},y_{p}\right) & = & \left\{\left(x_{p},y_{p}\right)\in R_{W}\;\subset \;R,\;\delta \in R_{+}\;|\;|x_{p}-x_{p}^{i}\left|+\right|y_{p}-y_{p}^{i}|\leq \delta ,\right.\\ & & \left.\;\mathrm{for}\;\mathrm{some}\;\left(x_{p}^{i},y_{p}^{i}\right)\in R_{W}\right\} \end{array}$$

with R the image plane. If the window $W_{i}\left(x_{p},y_{p}\right)$ is fixed inside the image plane, the computed speeds $u_{p}$ and $v_{p}$ are known as the optical flow. Using the differential method proposed by Lucas–Kanade [28], the computation of constant values of $u_{p}$ and $v_{p}$ in each small neighborhood $W_{i}\left(x_{p},y_{p}\right)$ can be implemented as the minimization of

$$\sum_{(x_{p},y_{p})\in W}Q{\left(x_{p},y_{p}\right)}^{2}{\left[\frac{\partial I_{1}}{\partial x_{p}}u_{p}+\frac{\partial I_{1}}{\partial y_{p}}v_{p}+\frac{\partial I_{1}}{\partial t}\right]}^{2}$$

with Q(x), a function giving more influence to constraints at the center of $W_{i}\left(x_{p},y_{p}\right)$ than those on the boundary. As reported in [29], this method is one of the most reliable.

**Figure 2:**
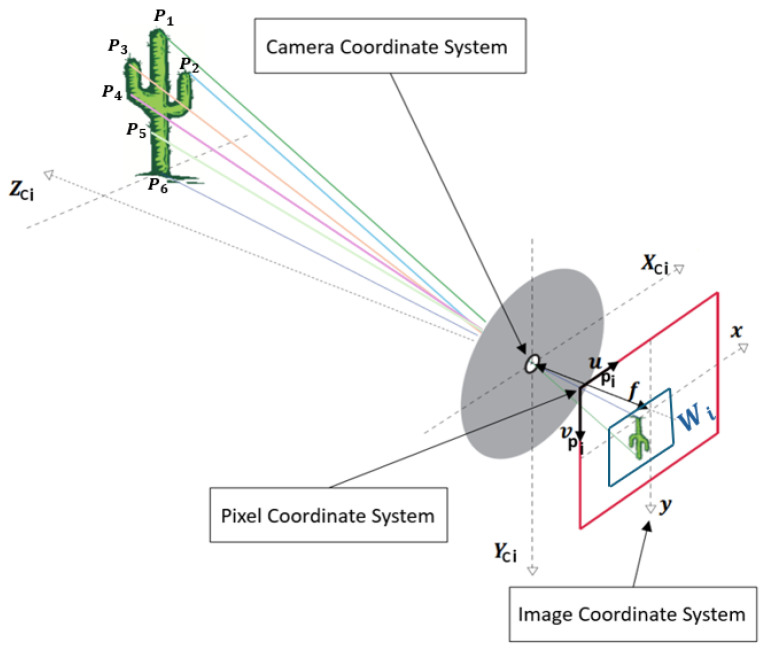
Pinhole camera principle [30].

Consider a camera onboard the aerial vehicle monitoring several characteristic points located at $P_{i}={\left[\begin{array}{ccc} x_{c}^{i} & y_{c}^{i} & z_{c}^{i} \end{array}\right]}^{⊤}$, as illustrated in Figure 2. It is worth mentioning that this Figure comes from [30] with slight additions. The velocity of each point relative to the camera is given by:

(10) $${\dot{P}}_{i}=-y_{3}\times P_{i}-V^{b}$$

From the perspective projection condition, it follows that the location of each characteristic point projects on the image plane as follows

(11) $$\begin{array}{ccc} x_{p}^{i} & = & f\frac{x_{c}^{i}}{z_{c}^{i}}\\ y_{p}^{i} & = & f\frac{y_{c}^{i}}{z_{c}^{i}} \end{array}$$

with *f* the focal length of the camera lens. Combining (10) and (11), one obtains

(12) $$\left[\begin{array}{c} u_{p}^{i}\\ v_{p}^{i} \end{array}\right]=\left[\begin{array}{c} -\frac{f}{z}u+\frac{x_{p}^{i}}{z}w+\frac{x_{p}^{i}y_{p}^{i}}{f}p-\left(f+\frac{{x_{p}^{i}}^{2}}{f}\right)q+y_{p}^{i}r\\ -\frac{f}{z}v+\frac{y_{p}^{i}}{z}w+\left(f+\frac{{y_{p}^{i}}^{2}}{f}\right)p-\frac{x_{p}^{i}y_{p}^{i}}{f}q-x_{p}^{i}r \end{array}\right]$$

the pixel velocity $\left(u_{p}^{i},v_{p}^{i}\right)$ registered by the camera due to the quadrotor motion.

Figure 2 shows that $z_{c}=z$. Using the differential method proposed by Lukas-Kanade to determine $u_{p}$ and $v_{p}$ in a region $W_{i}\left(x_{p},y_{p}\right)$, it is possible to obtain the quadrotor translational velocity as follows

(13) $$\left[\begin{array}{c} u\\ v \end{array}\right]=\left[\begin{array}{c} -\frac{z}{f}u_{p}+\frac{x_{p}}{f}w+z\frac{x_{p}y_{p}}{f^{2}}p-\frac{z}{f}\left(f+\frac{x_{p}^{2}}{f}\right)q+z\frac{y_{p}}{f}r\\ -\frac{z}{f}v+\frac{y_{p}}{f}w+\frac{z}{f}\left(f+\frac{y_{p}^{2}}{f}\right)p-z\frac{x_{p}y_{p}}{f^{2}}q-z\frac{x_{p}}{f}r \end{array}\right]$$

in an image region $W_{i}\left(x_{p},y_{p}\right)$ for some *i*.

Consider the following assumption,

**Assumption** **1.** *The optical flow is computed in a region $W_{i}$ that contains the pixel image origin; this is, $x_{p}=y_{p}=0$. Moreover, the quadrotor has a height controller that ensures $z\approx \bar{z}$ for some constant $\bar{z}$ and w≈0.*

Under Assumption 1, the Equation (13) becomes

(14) $$y_{4}=\left[\begin{array}{c} u_{p}\\ v_{p} \end{array}\right]=-H_{1}V^{b}+H_{2}y_{3}$$

with

$$H_{1}=\left[\begin{array}{ccc} \frac{f}{\bar{z}} & 0 & 0\\ 0 & \frac{f}{\bar{z}} & 0 \end{array}\right],\;H_{2}=\left[\begin{array}{ccc} 0 & -f & 0\\ f & 0 & 0 \end{array}\right]$$

a measurable signal.

#### 2.2.4. Height Sensor

Ultrasonic or laser devices to measure distance can be mounted on quadrotors. Hence, it is assumed that the following signals are also measured

(15) $$\begin{array}{ccc} y_{5} & = & z\\ y_{6} & = & w \end{array}$$

thus, the matrix $H_{1}$ is also measurable since it can be expressed as

$$H_{1}=\left[\begin{array}{ccc} \frac{f}{{\bar{y}}_{5}} & 0 & 0\\ 0 & \frac{f}{{\bar{y}}_{5}} & 0 \end{array}\right]$$

Finally, note that in terms of the measurable signals, the second equation in (7) can be expressed as

(16) $${\dot{V}}^{b}=gy_{2}^{⊤}e_{3}+y_{1}-s\left(y_{3}\right)V^{b}$$

## 3. Nonlinear Observer Design

**Figure 3:**
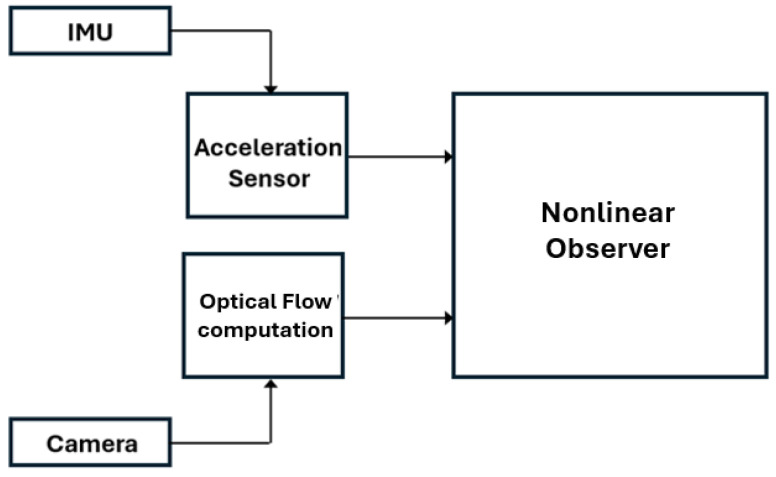
Sensor fusion.

Sensor fusion has become essential for solving mobile robotics state observation problems [31]. Better spatial and temporal coverage, robustness to sensor failures, and increased state observation accuracy are the main desirable properties of sensor fusion algorithms. This research article proposes a sensor fusion algorithm to estimate the quadrotor Cartesian velocity based on the Immersion and Invariance observer design technique proposed in [3]. The proposed fusion algorithm can be classified as a complementary fusion across domains [32,33]. Both considered sensors measure the same quantity in different domains and in acceleration and pixel velocity, and they work in a complementary configuration. Figure 3 illustrates schematically the proposed sensor fusion. The sensor fusion algorithm is designed in the deterministic nonlinear time-invariant framework.

The observer design method can be explained as follows. Consider the following non-linear, deterministic, time-invariant system [3].

(17) $$\begin{array}{cc} \dot{\eta } & =f_{1}\left(\eta \;,\;y\right)\\ \dot{y} & =f_{2}\left(\eta \;,\;y\right) \end{array}$$

where $\eta \;\in \;R\;\subset \;R^{n}$ and $y\;\in \;Y\;\subset \;R^{m}$ are the unmeasured and measured states, correspondingly.

**Definition** **1.** *The dynamic system*

(18) $$\dot{\hat{\eta }}=\phi \left(\hat{\eta }\;,\;y\right)$$

*with $\hat{\eta }\;\in \;R^{n}$, is a sensor fusion observer for the unmeasured state η if there exists a mapping $\beta \;:R^{n}\;\times R^{m}\;⟶\;R^{n}$ such that the manifold,*

(19) $$M=\left\{\left(\eta \;,\;\hat{\eta }\;,\;y\right)\;\subset \;R^{n}\;\times \;R^{n}\;\times \;R^{m}\;|\;\beta \left(\hat{\eta }\;,\;y\right)=\eta \right\}$$

*has the following properties,*

- *M is positively invariant;*
- *All trajectories of (17) and (18) that start in a neighborhood of M asymptotically converge to M.*

The design of the observer of the form given in Definition 1 requires additional properties on the mapping $\beta (\hat{\eta }\;,\;y)$, as stated in the following result.

**Theorem** **1.** *Consider the system (17). Assume that the vector fields $f_{1}\left(\eta \;,\;y\right)$ and $f_{2}\left(\eta \;,\;y\right)$ are forward complete and that there exist differentiable maps $\beta \;:\;R^{n}\;\times \;R^{m}\;⟶\;R^{n}$ such that*

*A1* *For all $\hat{\eta }$ and y the map $\beta (\hat{\eta }\;,\;y)$ satisfies,*
  $$det\left(\frac{\partial \beta }{\partial \hat{\eta }}\right)\;\neq \;0$$
*A2* *The dynamic system*
  (20) $$\tilde{\dot{\eta }}=f_{1}\left(\tilde{\eta }+\beta \left(\hat{\eta }\;,\;y\right)\;,\;y\right)-f_{1}\left(\beta \left(\hat{\eta }\;,\;y\right)\;,\;y\right)-\frac{\partial \beta }{\partial y}\left(f_{2}\left(\tilde{\eta }+\beta \left(\hat{\eta }\;,\;y\right)\;,\;y\right)-f_{2}\left(\beta \left(\hat{\eta }\;,\;y\right)\;,\;y\right)\right)$$
*has a (globally) asymptotically stable equilibrium at $\tilde{\eta }=0$ uniformly in $\hat{\eta }$ and y.*

*Then, the system (18) with,*

(21) $$\phi \left(\hat{\eta }\;,\;y\right)={\left(\frac{\partial \beta }{\partial \hat{\eta }}\right)}^{-1}\left(f_{1}\left(\beta \left(\hat{\eta }\;,\;y\right)\;,y\right)-\frac{\partial \beta }{\partial y}\;f_{2}\left(\beta \left(\hat{\eta }\;,\;y\right)\;,y\right)\right)$$

*is a (global) observer for (17).*

**Remark** **1.** *The result in Theorem 1 is a simplified version of the general observer design theory reported in [3]. The proof of Theorem 1 was reported in [17]. The sensor fusion characteristics of the observer in Definition 1 are as follows. Assume that two measurements $y_{1}$ and $y_{2}$ contain information on the nonmeasurable state η. Then, it is possible to define a function $\gamma (y_{1},y_{2})$ that fuses both measurements, and then the function $\beta (\hat{\eta },\gamma \left(y_{1},y_{2}\right))$ integrates the fusion to the observer design procedure.*

### Cartesian Velocity Observer

Here, the quadrotor Cartesian velocity observer is designed. The observer fuses all measurements described in Section 2.2. First, using all available measurements, the following measurements are tailored

(22) $$\begin{array}{ccc} {\bar{y}}_{1} & = & \left[\begin{array}{c} a_{x}^{b}\\ a_{y}^{b}\\ y_{6} \end{array}\right]={\bar{H}}_{1}V^{b}\\ {\bar{y}}_{4} & = & \left[\begin{array}{c} u_{p}\\ v_{p}\\ y_{6} \end{array}\right]={\bar{H}}_{2}V^{b}+{\bar{H}}_{3}y_{3} \end{array}$$

with

$${\bar{H}}_{1}=\left[\begin{array}{ccc} -\frac{\mu }{m} & 0 & 0\\ 0 & -\frac{\mu }{m} & 0\\ 0 & 0 & 1 \end{array}\right],\;{\bar{H}}_{2}=\left[\begin{array}{ccc} -\frac{f}{{\bar{y}}_{5}} & 0 & 0\\ 0 & -\frac{f}{{\bar{y}}_{5}} & 0\\ 0 & 0 & 1 \end{array}\right],\;{\bar{H}}_{3}=\left[\begin{array}{ccc} -f & 0 & 0\\ 0 & -f & 0\\ 0 & 0 & 0 \end{array}\right]$$

From the definition of the manifold (19), the observer error is defined as

(23) $${\tilde{V}}^{b}=V^{b}-\beta_{1}\left({\hat{V}}^{b}\;,\;\sigma \right)$$

with

(24) $$\dot{\sigma }=\gamma \left({\bar{y}}_{1},\;{\bar{y}}_{4}\right)=\frac{1}{\alpha_{1}+\alpha_{2}}\left(\alpha_{1}{\bar{y}}_{1}+\alpha_{2}{\bar{y}}_{4}\right)$$

with $\alpha_{1}$ and $\alpha_{2}$ scalar constants. Note that the function $\gamma ({\bar{y}}_{1},\;{\bar{y}}_{4})$ fuses the information of the same quantity, translational velocity, expressed in different domains, body axes acceleration ${\bar{y}}_{1}$, and optical flow ${\bar{y}}_{4}$. The scalars $\alpha_{1}$ and $\alpha_{2}$ modulate the fusion. The time derivative of the observer error ${\tilde{V}}^{b}$ reads as

(25) $${\dot{\tilde{V}}}^{b}={\dot{V}}^{b}-\frac{\partial \beta_{1}}{\partial {\hat{V}}^{b}}{\dot{\hat{V}}}^{b}-\frac{\partial \beta_{1}}{\partial \sigma }\gamma \left({\bar{y}}_{1},\;{\bar{y}}_{4}\right)$$

Using (16) and (22), one obtains

(26) $${\dot{\tilde{V}}}^{b}=gy_{2}^{T}e_{3}+y_{1}-S\left(y_{3}\right)V^{b}-\frac{\partial \beta_{1}}{\partial {\hat{V}}^{b}}{\dot{\hat{V}}}^{b}+\frac{1}{\alpha_{1}+\alpha_{2}}\frac{\partial \beta_{1}}{\partial \sigma }\left(\alpha_{1}{\bar{H}}_{1}V^{b}+\alpha_{2}\left({\bar{H}}_{2}V^{b}+{\bar{H}}_{3}y_{3}\right)\right)$$

Now, to express (26) in terms of the observer error, the Equation (23) is solved for $V^{b}$ and substituted in (26). Thus,

(27) $$\begin{array}{ccc} {\dot{\tilde{V}}}^{b} & = & gy_{2}^{T}e_{3}+y_{1}-S\left(y_{3}\right)\left({\tilde{V}}^{b}+\beta_{1}\left({\hat{V}}^{b}\;,\;\sigma \right)\right)-\frac{\partial \beta_{1}}{\partial {\hat{V}}^{b}}{\dot{\hat{V}}}^{b}\\ \; & \; & +\frac{1}{\alpha_{1}+\alpha_{2}}\frac{\partial \beta_{1}}{\partial \sigma }\left(\alpha_{1}{\bar{H}}_{1}\left({\tilde{V}}^{b}+\beta_{1}\left({\hat{V}}^{b}\;,\;\sigma \right)\right)+\alpha_{2}\left({\bar{H}}_{2}\left({\tilde{V}}^{b}+\beta_{1}\left({\hat{V}}^{b}\;,\;\sigma \right)\right)+{\bar{H}}_{3}y_{3}\right)\right) \end{array}$$

Defining

(28) $$\beta_{1}\left({\hat{V}}^{b},\sigma \right)={\hat{V}}^{b}+\Gamma \sigma$$

with Γ a matrix gain, the state observer dynamic can be defined as follows

(29) $${\dot{\hat{V}}}^{b}=gy_{3}^{T}e_{3}+y_{1}-S\left(y_{3}\right)\beta_{1}+\frac{1}{\alpha_{1}+\alpha_{2}}\Gamma \left(\alpha_{1}{\bar{H}}_{1}\beta_{1}\left({\hat{V}}^{b}\;,\;\sigma \right)+\alpha_{2}\left({\bar{H}}_{2}\beta_{1}\left({\hat{V}}^{b}\;,\;\sigma \right)+{\bar{H}}_{3}y_{3}\right)\right)$$

It is important to verify that the state observer dynamic depends only on available measurements and known parameters. Then, the following vector differential equation described the observer error dynamic

(30) $$\begin{array}{ccc} {\dot{\tilde{V}}}^{b} & = & -S\left(y_{3}\right){\tilde{V}}^{b}+\frac{1}{\alpha_{1}+\alpha_{2}}\Gamma \left(\alpha_{1}{\bar{H}}_{1}+\alpha_{2}{\bar{H}}_{2}\right){\tilde{V}}^{b} \end{array}$$

Hence, one has the following.

**Proposition** **1.** *Consider that Assumption 1 holds. Assume that the quadrotor is equipped with a set of sensors to measure $y_{i}$, i=1,⋯,6. Assume that the quadrotor flies over a surface with enough visual characteristics and there is a region $W_{i}$ containing the pixel location $x_{p}=y_{p}=0$, where the optical flow is constant. Then, there exist constants $\alpha_{1}$, $\alpha_{2}$ and a matrix Γ such that the observer dynamic (29) complimentarily fuses the available measurements and the observer error ${\tilde{V}}^{b}$ exponentially converges to zero.*

**Proof.**  The function $\gamma ({\bar{y}}_{1},{\bar{y}}_{4})$ in (24) performs the complementary fusion of the available measurements directly related to the quadrotor translational velocity. From this point, the observer design follows the lines of Theorem 1.

To analyze the observation error’s stability properties, consider the following Lyapunov function [2]

(31) $$V=\frac{1}{2}{\left({\tilde{V}}^{b}\right)}^{⊤}\Gamma_{1}{\tilde{V}}^{b}$$

with $\Gamma_{1}\in \;R^{3\times 3}$ a diagonal positive definite matrix. Thus, one has

(32) $$\lambda_{m}\left(\Gamma_{1}\right)∥{\tilde{V}}^{b}{∥}^{2}\leq V\leq \lambda_{M}\left(\Gamma_{1}\right){∥{\tilde{V}}^{b}∥}^{2}$$

with $\lambda_{m}\left(A\right)$ and $\lambda_{M}\left(A\right)$ the smallest and greatest eigenvalues of any matrix *A*.

The time derivative of (31) along the trajectories of the observer error dynamic (30) is given by

(33) $$\dot{V}={\left({\tilde{V}}^{b}\right)}^{⊤}\Gamma_{1}\left[-S\left(y_{3}\right){\tilde{V}}^{b}+\frac{1}{\alpha_{1}+\alpha_{2}}\Gamma \left(\alpha_{1}{\bar{H}}_{1}+\alpha_{2}{\bar{H}}_{2}\right){\tilde{V}}^{b}\right]$$

Since $S(y_{3})$ is a skew symmetric matrix, it follows that

(34) $$\dot{V}={\left({\tilde{V}}^{b}\right)}^{⊤}\left[\Gamma_{1}\frac{1}{\alpha_{1}+\alpha_{2}}\Gamma \left(\alpha_{1}{\bar{H}}_{1}+\alpha_{2}{\bar{H}}_{2}\right)\right]{\tilde{V}}^{b}$$

It is straightforward to verify that there exist $\alpha_{1}$, $\alpha_{2}$, Γ, and $\Gamma_{1}$ such that the matrix

(35) $$P=\left[\Gamma_{1}\frac{1}{\alpha_{1}+\alpha_{2}}\Gamma \left(\alpha_{1}{\bar{H}}_{1}+\alpha_{2}{\bar{H}}_{2}\right)\right]$$

is negative definite. Thus,

(36) $$\dot{V}\leq -\lambda_{m}\left(P\right){∥{\tilde{V}}^{b}∥}^{2}\leq -\frac{\lambda_{m}\left(P\right)}{\lambda_{M}\left(\Gamma_{1}\right)}V$$

and the proof is concluded. □

## 4. Results

This section presents a semi-realistic numerical simulation study to validate the theoretical developments of the previous section. First, the available measurements—the body axes acceleration and the optical flow—must be computed. Then, the proposed observer is implemented. It is essential to underscore that the Parrot Minidrone simulator provided by MATLAB-Simulink incorporates realistic quadrotor dynamics and sensor models. The works in [8,9,10] illustrate that the experimental implementation is straightforward after performing numerical simulations using this simulator; see also the work in [34], where semi-realistic simulations are performed.

The Parrot Minidrone’s physical characteristics and the camera specifications are summarized in Table 1.

### 4.1. Determination of the Parameter μ

Note that in Equation (24), two essential measurements to reconstruct the quadrotor velocity are ${\bar{y}}_{1}$ and ${\bar{y}}_{4}$. Table 1 shows that the quadrotor’s mass is available, but the parameter μ is not. Hence, the quadrotor follows a circular trajectory, recording, along the $0X^{b}$ axis, its acceleration $a_{x}^{b}$ and velocity *u* to determine μ from the following relationship

(37) $$\mu =-\frac{a_{x}^{b}}{u}m$$

**Figure 4:**
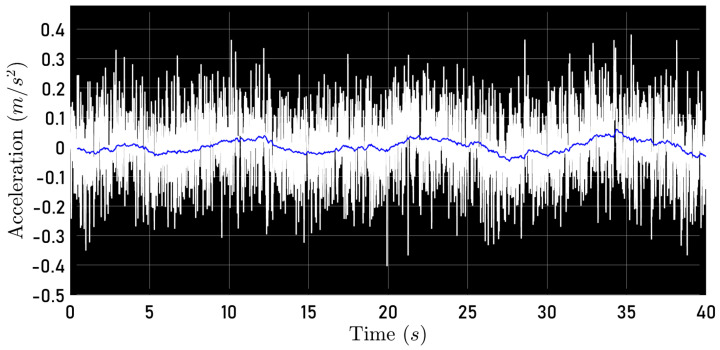
Measured acceleration $a_{x}^{b}$ and reconstructed acceleration ${\bar{a}}_{x}^{b}$.

It is important to highlight that to compute μ, the measured acceleration $a_{x}^{b}$ was filtered using a low-pass first-order filter, as recommended in [23]. Then, the value of μ was computed as the average value of the μ values obtained during the flight. Figure 4 shows (white line) the recorded acceleration $a^{b}$ and (blue line) the reconstructed acceleration; this is,

(38) $${\bar{a}}_{x}^{b}=-\frac{\mu }{m}u$$

Hence, for this quadrotor, one has μ=0.0035. The parameter μ is related to the blade’s aerodynamic profile and induced drag forces. This positive constant is known in helicopter literature as blade drag [23,35].

Note that this is an open-loop reconstruction so it is not expected that $a_{x}^{b}$ and ${\bar{a}}_{x}^{b}$ are exactly coincident. However, as reported in [17,23], this procedure gives an adequate approximation of μ.

### 4.2. Optical Flow Algorithm Design

The optical flow estimation algorithm is implemented in the Parrot Minidrone Competition simulation environment, specifically in the Image Processing subsystem in the Flight Control System block. The optical flow estimation algorithm is performed as follows: (a) The monocular camera information that arrives in the Y1UY2V format is transformed into the RGB format. (b) The image in RGB format is transformed to Grayscale and filtered using an FIR (Finite Response Impulse) filter represented as a 2D coefficient matrix or a pair of separable filter coefficient vectors. (c) The filtered image is used to estimate the optical flow, employing the Lucas–Kanade method. The block implementing the Lucas–Kanade method delivers the pixel displacement per frame as a complex number for each image’s pixel.

**Figure 5:**
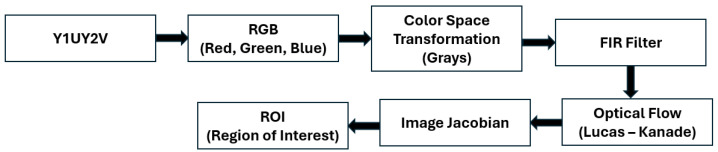
Optical flow block design.

These calculated displacements are multiplied by the corresponding number of frames per second (intrinsic parameter of the camera) to obtain the pixel velocity. Finally, the pixel velocities are subjected to statistical processing consisting of selecting a region of interest on the image plane, which, considering Assumption 1, corresponds to the image origin. Figure 5 shows a block diagram of the optical flow algorithm.

### 4.3. Quadrotor Trajectories

**Figure 6:**
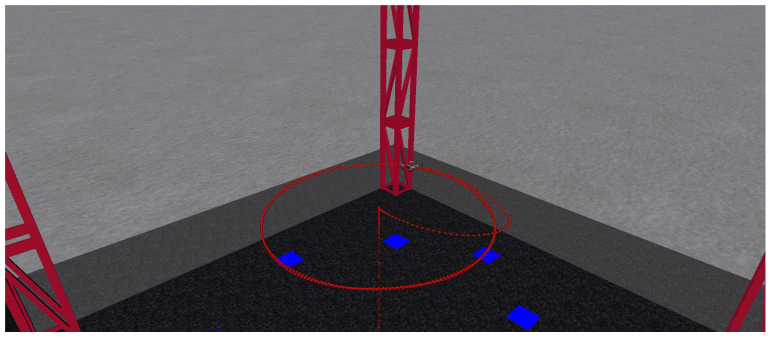
Aircraft tracking: circular-type trajectory.

**Figure 7:**
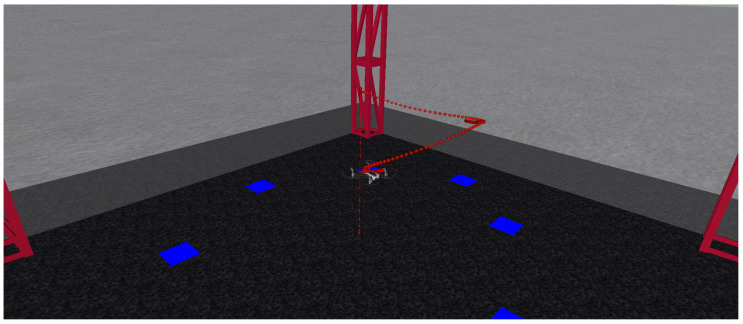
Aircraft tracking: square-type trajectory.

To test the proposed observer, two trajectories are considered. In the first one, the quadrotor tracks a circle while in the second one, the quadrotor visits two waypoints. Figure 6 shows the quadrotor’s path during the circle-tracking flight at a constant altitude. Figure 7 depicts the quadrotor’s path during the two-way point flight, which is at a constant altitude once again.

### 4.4. Measurements

**Figure 8:**
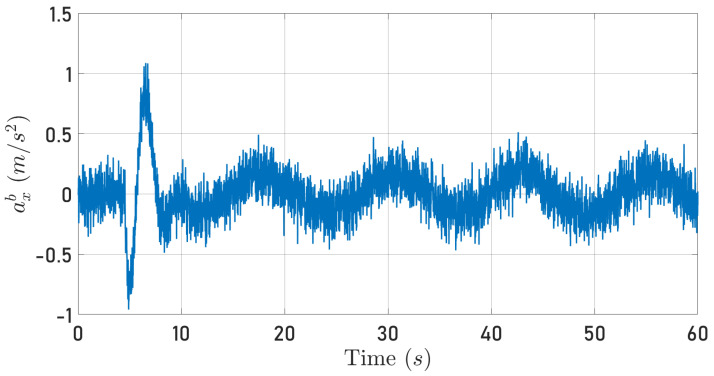
Specific force measured along the $0X^{b}$ axis while the quadrotor follows a circular trajectory.

**Figure 9:**
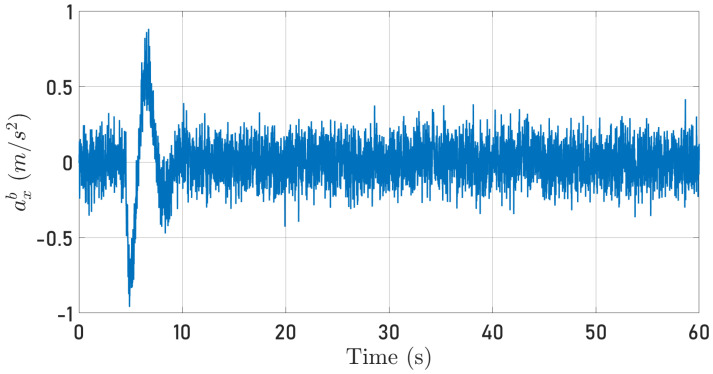
Specific force measured along the $0X^{b}$ axis while the quadrotor follows a square-type trajectory.

Note that the parameter μ is required to implement the proposed observer to reconstruct the quadrotor-specific force. The quadrotor acceleration is obtained directly from the IMU implemented in the simulator. Figure 8 and Figure 9 show the specific force along the $0X^{b}$ axis delivered by the IMU when the quadrotor tracks the circular and the square-type (the quadorotor moves first along the $0X^{b}$ axis and, 10 s after, moves along the $0Y^{b}$ axis) trajectories, respectively. It is essential to state that the IMU from Simulink is implemented considering that the accelerometers also measure the gravity force, contradicting Equation (4). Hence, the gravitational force is subtracted from the IMU’s accelerometer measurement to match (4).

**Figure 10:**
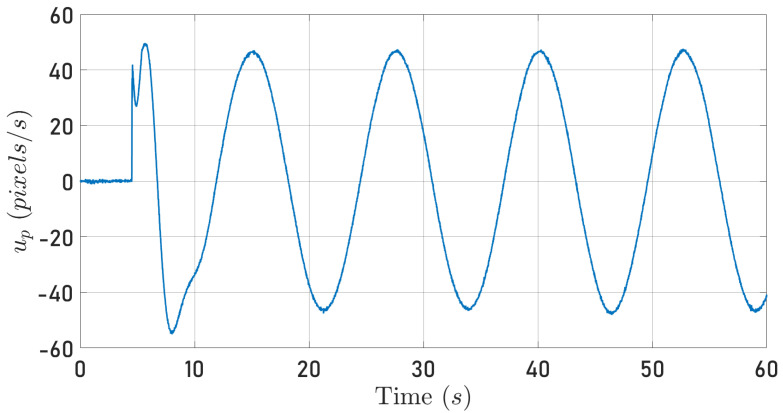
Computed optical flow along the $0X^{b}$ axis while the quadrotor tracks a circular trajectory.

**Figure 11:**
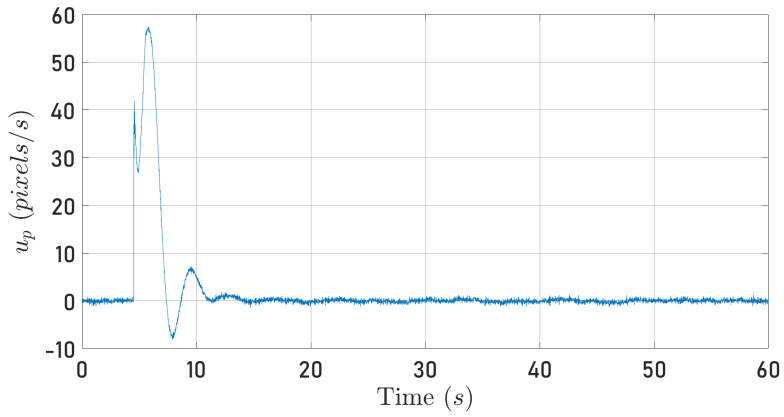
Computed optical flow along the $0X^{b}$ axis while the quadrotor tracks a square-type trajectory.

Figure 10 and Figure 11 depict the quadrotor optical flow along the $0X^{b}$ axis computed as described in Section 4.2 when the quadrotor follows the circular and square-type trajectories, respectively.

From Equation (22), it is clear that the signals plotted in Figure 8, Figure 9, Figure 10 and Figure 11 contain information about the translational velocity ${\tilde{V}}^{b}$. It is not evident to identify a relationship between the specific force and optical flow measurements. However, the proposed observer fuses the information and extracts the translational velocity ${\tilde{V}}^{b}$.

### 4.5. Observer Evaluation

The observer state dynamic described by Equation (29) is implemented inside the Parrot Mambo Minidrone simulator. The observed velocity

(39) $${\hat{V}}^{b}+\Gamma \sigma$$

is compared with the velocity computed by the internal algorithm of the Parrot Mambo Minidrone simulator, which employs a Kalman filter.

**Figure 12:**
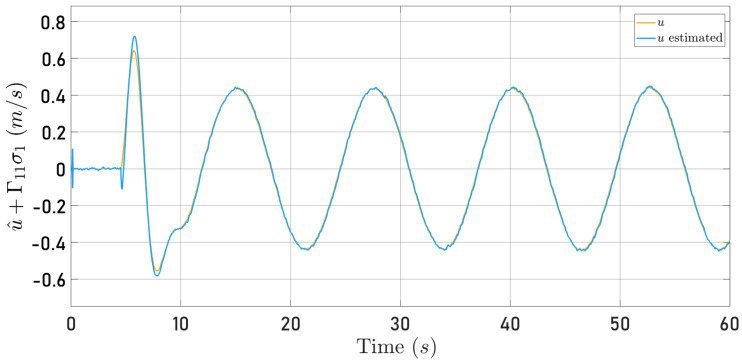
Observed speed $\hat{u}+\Gamma_{11}\sigma_{1}$ (blue line) and speed computed by the Parrot Mambo simulator algorithm *u* (yellow line).

**Figure 13:**
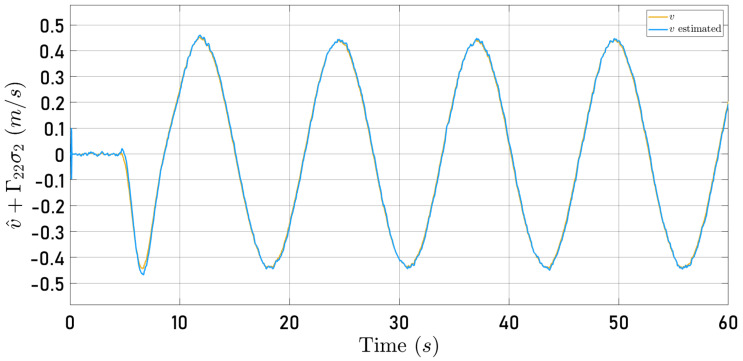
Observed speed $\hat{v}+\Gamma_{22}\sigma_{2}$ (blue line) and speed computed by the Parrot Mambo simulator algorithm *v* (yellow line).

Figure 12 and Figure 13 show the time history of the observed velocity along the $0X^{b}$ and $0Y^{b}$ axes, respectively, together with the speeds computed by the internal algorithm of the Parrot Mambo Minidrone simulator when the quadrotor follows the circular trajectory. The observer gain matrices were fixed as follows

$$\begin{array}{c} \Gamma =\left[\begin{array}{ccc} 6 & 0 & 0\\ 0 & 6 & 0\\ 0 & 0 & -1 \end{array}\right]\;;\;\Gamma_{1}=\left[\begin{array}{ccc} 1 & 0 & 0\\ 0 & 1 & 0\\ 0 & 0 & 1 \end{array}\right] \end{array}$$

The scalar constants $\alpha_{1}$ and $\alpha_{2}$ were fixed at the following values

(40) $$\alpha_{1}=-13\;;\;\alpha_{2}=0.4$$

**Figure 14:**
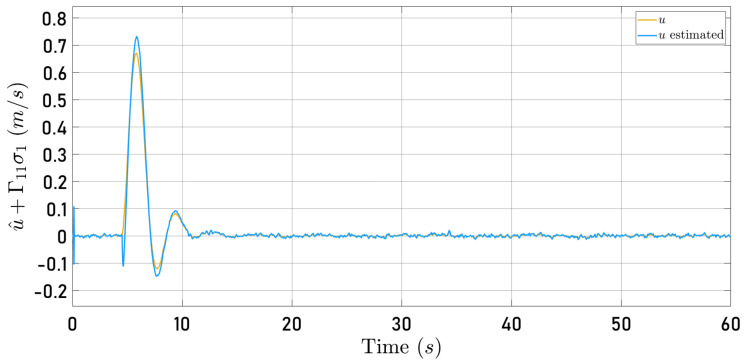
Observed speed $\hat{u}+\Gamma_{11}\sigma_{1}$ (blue line) and speed computed by the Parrot Mambo simulator algorithm *u* (yellow line).

**Figure 15:**
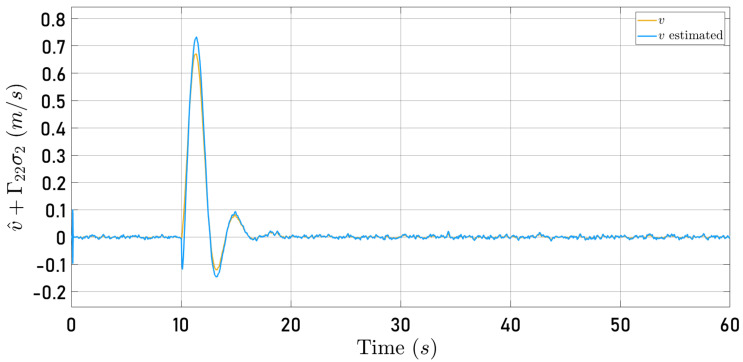
Observed speed $\hat{v}+\Gamma_{22}\sigma_{2}$ (blue line) and speed computed by the Parrot Mambo simulator algorithm *v* (yellow line).

Now, Figure 14 and Figure 15 present the observer error behavior when the quadrotor follows the square-type trajectory. The speeds computed by the Parrot Mambo Minidrone algorithm are also shown.

Figure 12, Figure 13, Figure 14 and Figure 15 illustrate that the selected observer gains performed adequately to identify both translational speeds.

**Figure 16:**
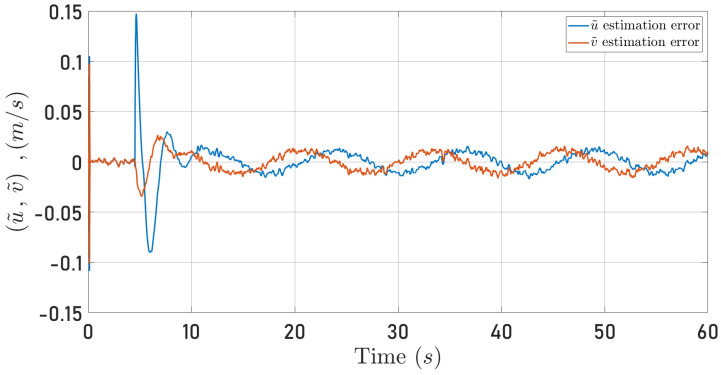
Observed speeds errors for the circular trajectory. $\tilde{u}$ (blue line), $\tilde{v}$ (red line).

**Figure 17:**
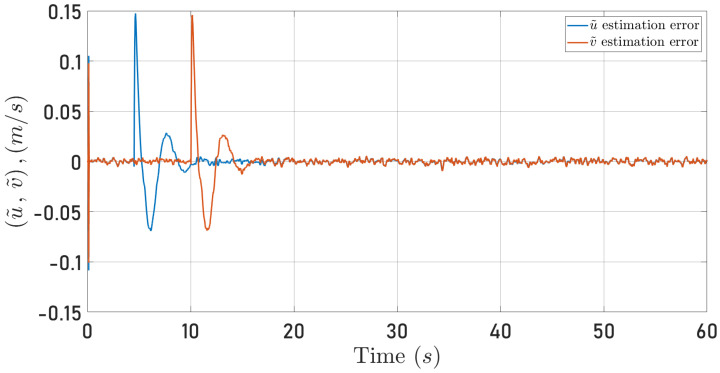
Observed speeds errors for the square-type trajectory. $\tilde{u}$ (blue line), $\tilde{v}$ (red line).

Figure 16 and Figure 17 present the observation errors to examine the proposed algorithm’s performance more closely. Note that the observation errors only converge to a neighborhood of zero. This behavior results from the semi-realistic simulation that includes noise in the measurements. However, the strong result in Proposition 1 hints at this observer behavior.

**Remark** **2.** *Under mild assumptions, consider that measurement noise enters into the translational velocity dynamics (16), as follows*

(41) $${\dot{V}}^{b}=gy_{2}^{⊤}e_{3}+y_{1}-s\left(y_{3}\right)V^{b}+\delta \left(t\right)$$

*with $\delta \left(t\right)\leq \bar{\delta }$ a bounded time variant disturbance modeling the noise in all measurements. Then, the observer error dynamic becomes*

(42) $$\begin{array}{ccc} {\dot{\tilde{V}}}^{b} & = & -S\left(y_{3}\right){\tilde{V}}^{b}+\frac{1}{\alpha_{1}+\alpha_{2}}\Gamma \left(\alpha_{1}{\bar{H}}_{1}+\alpha_{2}{\bar{H}}_{2}\right){\tilde{V}}^{b}-\delta \left(t\right) \end{array}$$

*Now, from the analysis in Proposition 1, one obtains*

(43) $$\begin{array}{ccc} \dot{V} & \leq & -\lambda_{m}\left(P\right)∥{\tilde{V}}^{b}{∥}^{2}+\lambda_{M}\left(\Gamma_{1}\right)\bar{\delta }∥{\tilde{V}}^{b}∥\\ \; & = & -\left(1-\sigma \right)\lambda_{m}\left(P\right)∥{\tilde{V}}^{b}{∥}^{2}-\sigma \lambda_{m}\left(P\right)∥{\tilde{V}}^{b}{∥}^{2}+\lambda_{M}\left(\Gamma_{1}\right)\bar{\delta }∥{\tilde{V}}^{b}∥ \end{array}$$

*with 0<σ<1. Finally,*

(44) $$\begin{array}{ccc} \dot{V} & \leq & -\left(1-\sigma \right)∥\lambda_{m}\left(P\right){\tilde{V}}^{b}{∥}^{2},\;\forall \; \end{array}∥{\tilde{V}}^{b}∥\geq \frac{\lambda_{M}\left(\Gamma_{1}\right)\bar{\delta }}{\sigma \lambda_{m}\left(P\right)}$$

*Hence, from Lemma 9.2 in [2], it follows that the observer error is ultimately bounded as observed in Figure 16. Note in (44), that the ultimate bound depends on the noise level modeled by $\bar{\delta }$.*

*A more challenging problem where the proposed observer design method may fail is the case where the noise enters the translational velocity dynamic as follows*

(45) $${\dot{V}}^{b}=gy_{2}^{⊤}e_{3}+y_{1}-s\left(y_{3}\right)V^{b}+\delta_{1}\left(t\right)V^{b}+\delta_{2}\left(t\right)$$

*with $\delta_{1}\left(t\right)\leq {\bar{\delta }}_{1}$ and $\delta_{2}\left(t\right)\leq {\bar{\delta }}_{2}$ bounded time variant disturbances.*

### 4.6. Observer Gains

A nonlinear time-varying model describes the observer error dynamics (see Equation (30)). Thus, determining adequate observer gains is a complex task; however, with some mild assumptions, at least locally, it is possible to determine a suitable observer gains combination. For example, assume that $y_{3}\approx 0$; then, the observer error dynamics reduces to

(46) $$\begin{array}{ccc} {\dot{\tilde{V}}}^{b} & = & \left[\begin{array}{ccc} \frac{-\gamma_{1}\left(\alpha_{1}\frac{\mu }{m}+\alpha_{2}\frac{f}{{\bar{y}}_{5}}\right)}{\alpha_{1}+\alpha_{2}} & 0 & 0\\ 0 & \frac{-\gamma_{2}\left(\alpha_{1}\frac{\mu }{m}+\alpha_{2}\frac{f}{{\bar{y}}_{5}}\right)}{\alpha_{1}+\alpha_{2}} & 0\\ 0 & 0 & 2\gamma_{3} \end{array}\right]{\tilde{V}}^{b} \end{array}$$

Hence, the following Eigenvalues locally shape the observer error dynamics

(47) $$\begin{array}{ccc} \lambda_{1} & = & \frac{-\gamma_{1}\left(\alpha_{1}\frac{\mu }{m}+\alpha_{2}\frac{f}{{\bar{y}}_{5}}\right)}{\alpha_{1}+\alpha_{2}}\\ \lambda_{2} & = & \frac{-\gamma_{2}\left(\alpha_{1}\frac{\mu }{m}+\alpha_{2}\frac{f}{{\bar{y}}_{5}}\right)}{\alpha_{1}+\alpha_{2}}\\ \lambda_{3} & = & 2\gamma_{3} \end{array}$$

**Figure 18:**
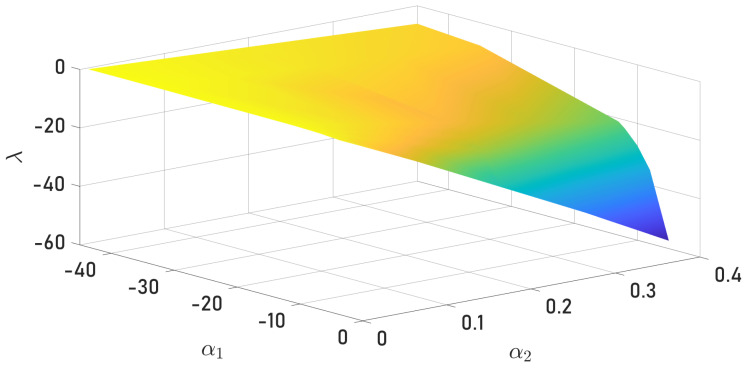
Eigenvalues for different combinations of gains $\alpha_{1}$ and $\alpha_{2}$.

From Equation (47), the selection $\gamma_{3}$ follows trivially. Now, assuming that $\gamma_{1}=\gamma_{2}$, the combination of $\gamma_{1}\;,\;\alpha_{1}$ and $\alpha_{2}$ was selected as follows. For a fixed value of $\gamma_{1}$, the Eigenvalue $\lambda =\lambda_{1}$ was computed considering intervals for $\gamma_{1}$ and $\gamma_{2}$ that give a negative value, as illustrated in Figure 18. It is important to remember that a negative Eigenvalue will not guarantee an adequate observation of the quadrotor velocity due to numerical problems in the simulation.

**Figure 19:**
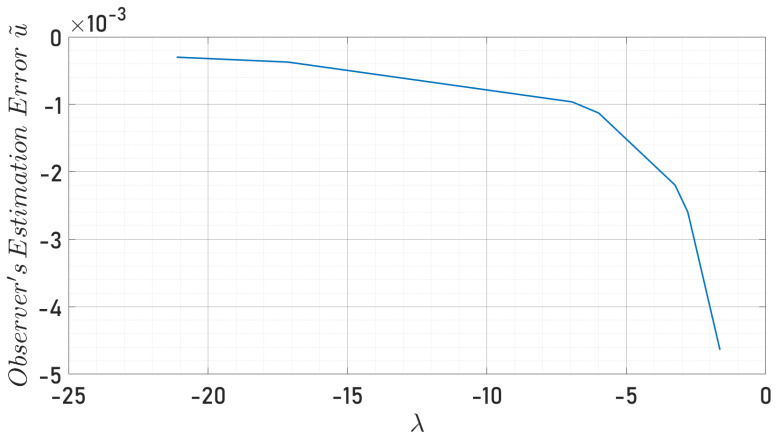
Eigenvalues vs. observer’s estimation error in the $0X^{b}$ axis.

In Figure 18, one can observe the optimal values region from the scalar constants, as well as the Eigenvalues, described previously, that were estimated with precision and accuracy. The displayed chromatic variety denotes the set of Eigenvalues λ, according to the different values that the scalar constants $\alpha_{1}\;,\;\alpha_{2}$ can take. The last result Figure 19 shows the estimation errors analyzed only with respect to the *x*-axis because analogically, the *y*-axis presents the same behavior. As expected, increasing the value of λ decreases the observer error until the numerical errors appear at a specific value of λ, where the observer error diverges.

## 5. Conclusions

This article proposed a novel sensor fusion observation algorithm to observe the translational velocity using available measurements from onboard sensors for a quadrotor. Besides the successful implementation of the designed observation algorithm, the main contributions are listed next:

- The designed observer can estimate the linear speeds of the aircraft for different trajectories with precision.
- The optical flow was obtained without using image features or patterns compared to other research works.
- The application of Lyapunov’s theory through a correct proposed Lyapunov function demonstrates asymptotic convergence to zero of the nonlinear observer error.
- As mentioned initially, this article was intended to compensate for the overall information loss in indoor flights by observing the vehicle’s translational velocities. The observed velocity is precise enough so that the main objective has been successfully fulfilled.
- As previously evoked in the introduction, this research project surpassed the performance of the observer proposed in [17], mainly in flight trajectories, in which, at certain moments, the MAV does not make any displacements, remaining in stationary or hover flight, thanks to the incorporation of the *Optical Flow* into the observation algorithm.

## Figures and Tables

**Table 1:** Drone’s physical characteristics and camera specifications.

Dimensions	(13.2 × 13.2 × 4.1 ) cm.
Weight	(63) g.
Motor type	Brushless (4).
Flight time	(8) min.
Camera	(Monocular / Vertical).
Shutter speed	(60) fps.
Resolution	(120 × 160) px.
Focal length	(1) mm.

## Data Availability

Simulation files are available upon request to the first or second authors.

## References

[1] Lyapunov A.M. (1992). The general problem of the stability of motion. Int. J. Control..

[2] Khalil H.K. (2002). Control of Nonlinear Systems.

[3] Astolfi A., Karagiannis D., Ortega R. (2008). Nonlinear and Adaptive Control with Applications.

[4] Van der Schaft A. (2016). L2-Gain and Passivity Techniques in Nonlinear Control.

[5] Sasiadek J., Hartana P. Sensor fusion for navigation of an autonomous unmanned aerial vehicle. Proceedings of the IEEE International Conference on Robotics and Automation (ICRA’04).

[6] Jetto L., Longhi S., Venturini G. (1999). Development and experimental validation of an adaptive extended Kalman filter for the localization of mobile robots. IEEE Trans. Robot. Autom..

[7] Harriman D.W., Harrison J.C. (1986). Gravity-induced errors in airborne inertial navigation. J. Guid. Control. Dyn..

[8] Noordin A., Mohd Basri M.A., Mohamed Z. (2023). Adaptive PID Control via Sliding Mode for Position Tracking of Quadrotor MAV: Simulation and Real-Time Experiment Evaluation. Aerospace.

[9] Okasha M., Kralev J., Islam M. (2022). Design and Experimental Comparison of PID, LQR and MPC Stabilizing Controllers for Parrot Mambo Mini-Drone. Aerospace.

[10] Rubio Scola I., Guijarro Reyes G.A., Garcia Carrillo L.R., Hespanha J.P., Burlion L. (2021). A Robust Control Strategy With Perturbation Estimation for the Parrot Mambo Platform. IEEE Trans. Control. Syst. Technol..

[11] Zhu C., Chen J., Zhang H. (2023). Attitude Control for Quadrotors Under Unknown Disturbances Using Triple-Step Method and Nonlinear Integral Sliding Mode. IEEE Trans. Ind. Electron..

[12] Naseer F., Ullah G., Siddiqui M.A., Jawad Khan M., Hong K.S., Naseer N. Deep Learning-Based Unmanned Aerial Vehicle Control with Hand Gesture and Computer Vision. Proceedings of the 2022 13th Asian Control Conference (ASCC).

[13] Nascimento T., Saska M. (2021). Embedded fast nonlinear model predictive control for micro aerial vehicles. J. Intell. Robot. Syst..

[14] McGuire K., de Croon G., de Wagter C., Remes B., Tuyls K., Kappen H. Local histogram matching for efficient optical flow computation applied to velocity estimation on pocket drones. Proceedings of the 2016 IEEE International Conference on Robotics and Automation (ICRA).

[15] Hoang M.L., Carratù M., Paciello V., Pietrosanto A. (2023). Fusion Filters between the No Motion No Integration Technique and Kalman Filter in Noise Optimization on a 6DoF Drone for Orientation Tracking. Sensors.

[16] Benzemrane K., Damm G., Santosuosso G. Nonlinear adaptive observer for Unmanned Aerial Vehicle without GPS measurements. Proceedings of the 2009 European Control Conference (ECC).

[17] Gómez-Casasola A., Rodríguez-Cortés H. (2022). Scale Factor Estimation for Quadrotor Monocular-Vision Positioning Algorithms. Sensors.

[18] Borup K.T., Fossen T.I., Johansen T.A. (2016). A nonlinear model-based wind velocity observer for unmanned aerial vehicles. IFAC-PapersOnLine.

[19] Hosen J., Helgesen H.H., Fusini L., Fossen T.I., Johansen T. A Vision-aided Nonlinear Observer for Fixed-wing UAV Navigation. Proceedings of the AIAA Guidance, Navigation, and Control Conference.

[20] He Y., Wang D., Huang F., Zhang R., Min L. (2023). Aerial-Ground Integrated Vehicular Networks: A UAV-Vehicle Collaboration Perspective. IEEE Trans. Intell. Transp. Syst..

[21] Kalenberg K., Müller H., Polonelli T., Schiaffino A., Niculescu V., Cioflan C., Magno M., Benini L. (2024). Stargate: Multimodal Sensor Fusion for Autonomous Navigation On Miniaturized UAVs. IEEE Internet Things J..

[22] Mahony R., Kumar V., Corke P. (2012). Multirotor Aerial Vehicles: Modeling, Estimation, and Control of Quadrotor. IEEE Robot. Autom. Mag..

[23] Leishman R.C., Macdonald J.C., Beard R.W., McLain T.W. (2014). Quadrotors and Accelerometers: State Estimation with an Improved Dynamic Model. IEEE Control. Syst. Mag..

[24] Ma Y., Soatto S., Košecká J., Sastry S. (2004). An Invitation to 3-D Vision: From Images to Geometric Models.

[25] Xie N., Lin X., Yu Y. Position estimation and control for quadrotor using optical flow and GPS sensors. Proceedings of the 2016 31st Youth Academic Annual Conference of Chinese Association of Automation (YAC).

[26] Weiss L., Sanderson A., Neuman C. (1987). Dynamic sensor-based control of robots with visual feedback. IEEE J. Robot. Autom..

[27] Wu Y. (2001). *Optical Flow and Motion Analysis*; Advanced Computer Vision Notes Series 6. http://www.eecs.northwestern.edu/~yingwu/teaching/EECS432/Notes/optical_flow.pdf.

[28] Lucas B.D., Kanade T. An iterative image registration technique with an application to stereo vision. Proceedings of the IJCAI’81: 7th International Joint Conference on Artificial Intelligence.

[29] Barron J.L., Fleet D.J., Beauchemin S.S. (1994). Performance of optical flow techniques. Int. J. Comput. Vis..

[30] Spagl M. (2021). Optische Positions-und Lagebestimmung einer Drohne im Geschlossenen Raum. Master’s Thesis.

[31] Mitchell H.B. (2007). Multi-Sensor Data Fusion: An Introduction.

[32] Durrant-Whyte H.F. (1988). Sensor models and multisensor integration. Int. J. Robot. Res..

[33] Boudjemaa R., Forbes A. (2004). *Parameter Estimation Methods in Data Fusion*; NPL Report CMSC 38/04. https://eprintspublications.npl.co.uk/2891/1/CMSC38.pdf.

[34] Noordin A., Mohd Basri M.A., Mohamed Z. (2022). Position and attitude tracking of MAV quadrotor using SMC-based adaptive PID controller. Drones.

[35] Bramwell A.R.S., Balmford D., Done G. (2001). Bramwell’s Helicopter Dynamics.

